# (Z)3,4,5,4′-trans-tetramethoxystilbene, a new analogue of resveratrol, inhibits gefitinb-resistant non-small cell lung cancer via selectively elevating intracellular calcium level

**DOI:** 10.1038/srep16348

**Published:** 2015-11-06

**Authors:** Xing-Xing Fan, Xiao-Jun Yao, Su Wei Xu, Vincent Kam-Wai Wong, Jian-Xing He, Jian Ding, Wei-Wei Xue, Tahira Mujtaba, Francesco Michelangeli, Min Huang, Jun Huang, Da-Kai Xiao, Ze-Bo Jiang, Yan-Ling Zhou, Richard Kin-Ting Kam, Liang Liu, Elaine Lai-Han Leung

**Affiliations:** 1 State Key Laboratory of Quality Research in Chinese Medicine/Macau Institute For Applied Research in Medicine and Health, Macau University of Science and Technology, Macau (SAR), China; 2 Guangzhou Institute of Respiratory Disease, State Key Laboratory of Respiratory Disease, The 1^st^ Affiliated Hospital of Guangzhou Medical College, Guangzhou, China; 3 State Key Laboratory of Drug Research, Shanghai Institute of Materia Medica, Chinese Academic of Sciences, Shanghai, China; 4 Department Chemical Pathology, Faculty of Medicine, the Chinese University of Hong Kong, Hong Kong (SAR), China; 5 Innovative Drug Research Centre, Chongqing University, Chongqing, China; 6 Department of Biological Sciences, University of Chester, Chester, United Kingdom

## Abstract

Calcium is a second messenger which is required for regulation of many cellular processes. However, excessive elevation or prolonged activation of calcium signaling would lead to cell death. As such, selectively regulating calcium signaling could be an alternative approach for anti-cancer therapy. Recently, we have identified an effective analogue of resveratrol, (Z)3,4,5,4′-trans-tetramethoxystilbene (TMS) which selectively elevated the intracellular calcium level in gefitinib-resistant (G-R) non-small-cell lung cancer (NSCLC) cells. TMS exhibited significant inhibitory effect on G-R NSCLC cells, but not other NSCLC cells and normal lung epithelial cells. The phosphorylation and activation of EGFR were inhibited by TMS in G-R cells. TMS induced caspase-independent apoptosis and autophagy by directly binding to SERCA and causing endoplasmic reticulum (ER) stress and AMPK activation. Proteomics analysis also further confirmed that mTOR pathway, which is the downstream of AMPK, was significantly suppressed by TMS. JNK, the cross-linker of ER stress and mTOR pathway was significantly activated by TMS. In addition, the inhibition of JNK activation can partially block the effect of TMS. Taken together, TMS showed promising anti-cancer activity by mediating calcium signaling pathway and inducing apoptosis as well as autophagy in G-R NSCLC cells, providing strategy in designing multi-targeting drug for treating G-R patients.

Non-small–cell lung cancer (NSCLC) is the most common type of lung cancer which is the leading cause of cancer-related death^1^. Most NSCLC patients are initially responsive to chemotherapy, but drug resistance ultimately occurs and leads to cancer recurrence and poor prognosis^2^. Molecular targeting therapy for lung cancer was first FDA-approved in 2004 which brings new insights and enriches the strategies of therapy for lung cancer^3^. The pioneer example, gefitinib, which is a tyrosine kinase inhibitor (TKI) of epidermal growth factor receptor (EGFR), can specifically block the activation of EGFR by binding to its ATP binding pocket, resulting in EGFR kinase inhibition^4^. Patients with EGFR activating mutation response well to gefitinib treatment at the beginning, however, further mutation on EGFR or alternative pathway would soon emerge within 12 months after the treatment of gefitinib and finally lead to drug resistance^5^. Therefore, novel anti-cancer agents or treatment strategies are deeply required for patients, especially for the TKI-resistant patients.

Resveratrol has been demonstrated with multiple promising pharmacological activities for longevity, treatment of heart disease, diabetes and cancer^6^. Resveratrol is a polyphenol which wildly exists in grapes and red wine. The investigation of ‘French Paradox’ which describes improved cardiovascular outcomes despite a high-fat diet in French people opens the study of resveratrol in many disorders and diseases^7^,^8^,^9^,^10^. It’s anti-cancer effect has been well demonstrated in various types of cancer by regulating cell division, growth, angiogenesis and metastasis^11^. In lung cancer, it has been reported that resveratrol induces premature senescence in lung cancer cells (A549 and H460 cells) via induction of NAPDH oxidase-5 (Nox5) expression^12^, resulting in inhibition of proliferation and survival^13^. However, until now, only one analogue of resveratrol, DMU-212 (Chemical structure as shown in Fig. 1a), has been tested in the pre-clinical stage for anti-cancer therapy, which has been shown to have very strong anti-cancer activity in multiply cancers, like colon^14^,^15^ and ovarian cancer^16^. However, to our knowledge, there is no report and investigation of the effect of resveratrol or its derivatives on gefitinib resistant (G-R) NSCLC.

In this study, we have identified an effective resveratrol derivative, TMS, which can selectively inhibit the growth of G-R NSCLC cells whereas it is relatively non-toxic to normal lung epithelial cells. Our study has demonstrated that TMS is a potential new anti-cancer agent particularly for G-R NSCLC patient as it shows selective inhibiting activity on G-R NSCLC. In addition, TMS exhibits anti-cancer activity different from resveratrol and DMU-212, which provides a new drug of choice for further therapeutic development.

## Results

### TMS exhibits selective cytotoxic effect towards G-R NSCLC cells

**Figure 1:**
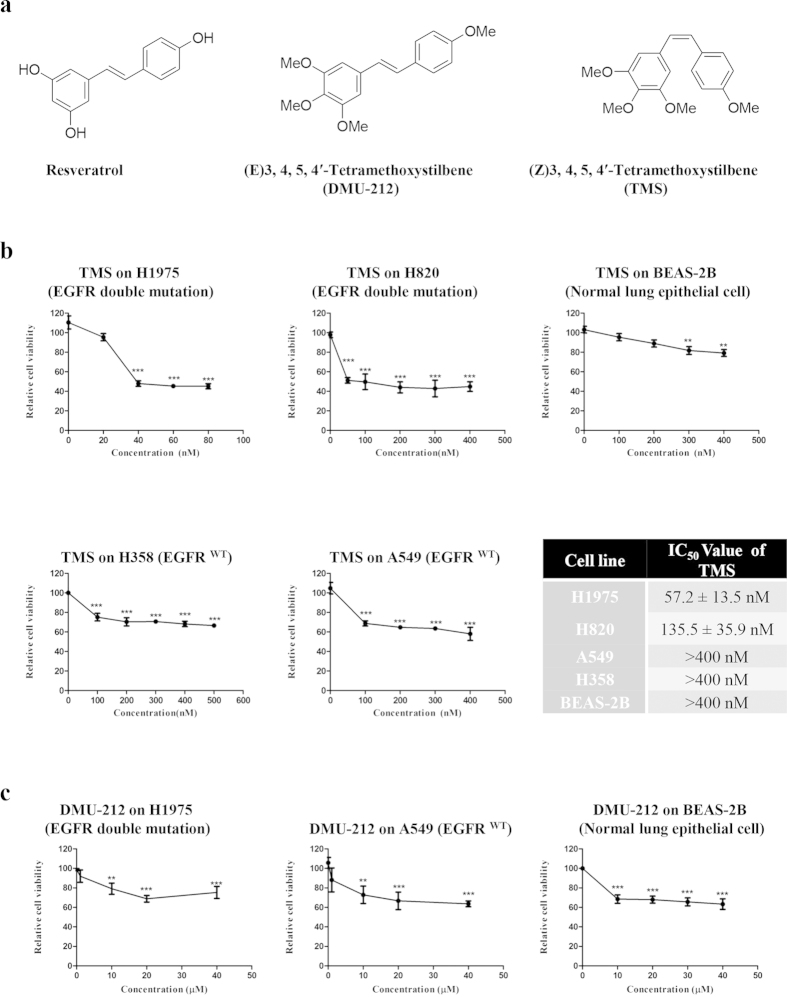
TMS showed selectivity on G-R NSCLC cells. (**a**) The chemical structures of resveratrol and its two derivatives: (E)3,4,5,4′-Tetramethoxystilbene (DMU-212) and (Z)3,4,5,4′-Tetramethoxystilbene (TMS). (**b**) The dose response curve and IC_50_ value of TMS on NSCLC cell lines and BEAS-2B normal lung epithelial cell line. (**c**) The dose response curve of DMU-212 on NSCLC cells and BEAS-2B cells. Results were expressed as mean ± S.E. (*p < 0.05, **p < 0.01, ***p < 0.001).

The effect of TMS on cell growth was investigated with four NSCLC cell lines, H1975, H820, A549, H358 and one normal lung epithelial cell line (BEAS-2B). Among the four NSCLC cell lines, they have different EGFR genetic mutations, H1975 harbors L858R and T790M double mutation on EGFR, H820 harbors exon 19 in frame deletion and T790M double mutation on EGFR, while A549 and H358 have wild-type EGFR. After culturing and treating the cells with a wide concentration of range of TMS for 24 hrs, the viability of cells was determined using MTT assay. As shown in Fig. 1b, TMS was more effective in inhibiting the growth of G-R (H1975 and H820) NSCLC cells than the other NSCLC cells. Most interestingly, the inhibitory effect of TMS on normal lung epithelial cells (BEAS-2B) was the weakest. Even using 10 fold higher concentration of the IC_50_ value of H1975 to treat BEAS-2B, there is still no significant inhibitory effect, implying low toxicity of TMS to normal lung epithelium.

Moreover, we have also compared the effect of TMS with DMU-212, which is another documented derivative of resveratrol currently on clinical trial, on the same NSCLC cells and normal lung epithelial cells. As shown in Fig. 1c, unlike TMS, DMU-212 didn’t show the discriminatory potential in G-R NSCLC cells or normal cells and the IC_50_ values (>40 μM) is much higher than that of TMS (57.2 nM). Taken together, TMS showed better and more specific inhibition effect on G-R NSCLC cells than DMU-212.

### TMS effectively induces caspase-independent apoptosis in G-R NSCLC cells

**Figure 2:**
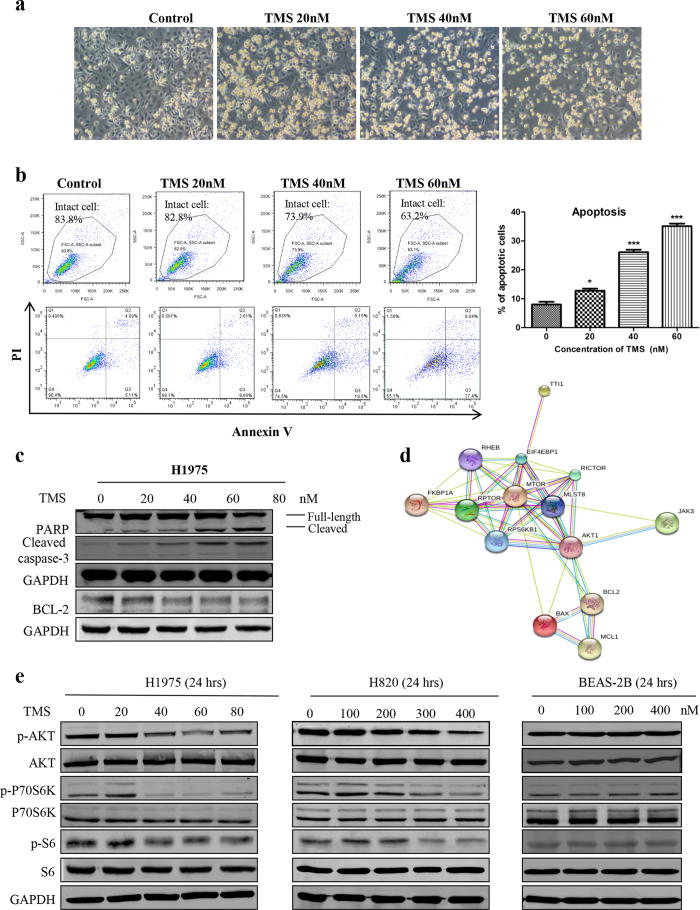
TMS significantly induced apoptosis in H1975 and suppressed the mTOR pathway. (**a**) The morphological changes of H1975 cells after treatment of TMS. (**b**) Flow cytometric analysis of the apoptosis level after TMS treatment. Statistical analysis result of b. (**c**) PARP and caspase-3 were cleaved and activated by TMS, while Bcl-2 was downregulated. (**d**) The result of proteomics showed that mTOR pathway was inhibited by TMS. (**e**) Western-blot result confirmed that TMS selectively inhibited mTOR pathway in G-R NSCLC cells H1975 and H820, but no effect on normal lung epithelial cells BEAS-2B. (*p < 0.05, **p < 0.01, ***p < 0.001).

To investigate whether TMS can induce apoptosis in G-R NSCLC cells, we measured apoptosis in H1975 after TMS treatment. Firstly, we examined the apoptotic effect of TMS by microscopic and flow cytometric analysis. As shown in Fig. 2a,b, both results revealed that TMS significantly induced apoptosis in H1975. For example, in control group, there were less than 10% of cells undergoing apoptosis (Q2+Q3). When cells were treated with 40 nM TMS, apoptotic cells increased to 24.65% and in the 60 nM TMS treatment group, 33.44% cells were undergoing apoptosis. Moreover, as shown in Fig. 2c, both PARP and caspase-3 were significantly cleaved and activated by TMS while Bcl-2 was gradually decreased.

To explore whether caspase activation is required for the apoptosis induced by TMS, pan-caspase inhibitor Z-VAD-FMK was applied to block caspase activities. Notably, treatment with pan-caspase inhibitor failed to block the apoptotic effect of TMS, suggesting that apoptosis induced by TMS is independent on caspase activation (Supplementary Figure 1). These results indicated that TMS significantly induced apoptosis in G-R cells through caspase-independent pathway.

### iTRAQ proteomic analysis demonstrated that mTOR pathway is involved in exerting the specific toxicity of TMS in G-R NSCLC cells

To identify the potential targets of TMS, protein lysate extracted from H1975 cells with or without TMS treatment were compared by iTRAQ labeling proteomics method. After digestion and labeling, proteins were subjected to the automated LC-MS/MS analysis. The information of signals was analyzed by MASCOT search engine and SwissProt 51.6 database. As shown in Table 1, when compared with the control group, totally there were 78 proteins down-regulated and 75 proteins up-regulated by TMS with more than 1.5 folds difference. To further deciphering the related signaling pathway of TMS, the regulated 153 proteins were inputted into Kyoto Encyclopedia of Genes and Genomes (KEGG) pathway database for bioinformatics analysis. Interestingly, as shown in Fig. 2d, twenty-two proteins were involved in key core mechanistic target of rapamycin (mTOR) pathway and its downstream ribosome biogenesis pathways. For example, mTOR, S6K, EIF4EBP1 and BSM1 were all significantly down-regulated, which suggested that protein synthesis was largely inhibited by TMS in H1975 cells.

To confirm the results of proteomics, we examined the effect of TMS on the expression level of the key members in the mTOR pathway. Figure 2e showed that both upstream and downstream members of mTOR pathway, such as AKT, P70S6 kinase and S6 kinase, were all significantly inhibited by TMS in both H1975 and H820 cells but not in normal lung BEAS-2B epithelial cells. Overall, the results of proteomics and western blot confirmed that mTOR pathway was involved in mediating the anti-cancer effect of TMS.

### TMS induces apoptosis and suppresses mTOR pathway by significantly increasing the cellular calcium level

**Figure 3:**
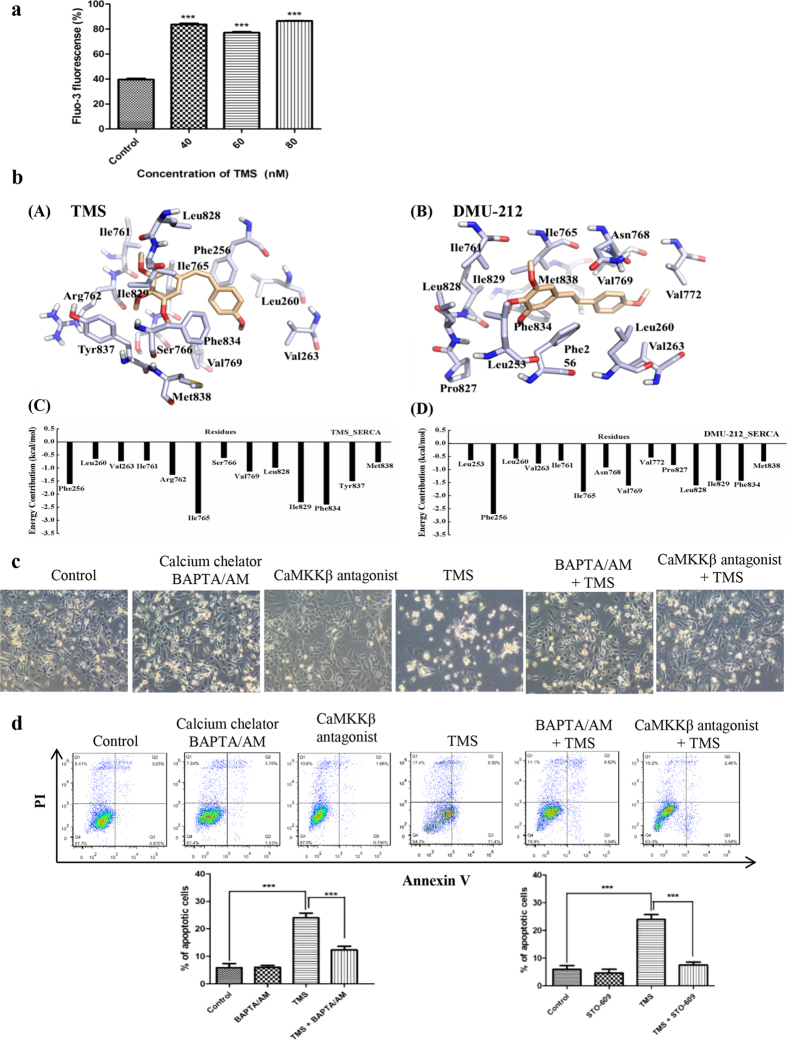
TMS regulated the [Ca^2+^] level in H1975 by directly targeting SERCA and inhibited the growth of cells. (**a**) TMS significantly elevated the [Ca^2+^] level after 2 hours treatment. (**b**) Modeling of the TMS and DMU-212 bound to the SERCA. Binding mode and per-residue interaction energy of SERCA with TMS (**A,C**) and DMU-212 (**B,D**) were presented. Residues with the absolute energy contribution ≥0.5 kcal/mol are shown. (**c**) TMS significantly induced the ROS generation in H1975. (**d**) The increase of intracellular [Ca^2+^] level was required for TMS to inhibit the growth of H1975 cells. Calcium chelator (BM) and CaMKKβ inhibitor (STO-609) remarkably inhibited the apoptosis induced by TMS. (*p < 0.05, **p < 0.01, ***p < 0.001).

To explore the upstream mechanism of TMS that drove the effect of induction of apoptosis and suppression of the mTOR pathway, firstly we measured the reactive oxygen species (ROS) levels. However, no significant change in ROS was observed (Supplementary Figure 2), which suggested that oxidative stress was not involved in mediating TMS action. Since mTOR could be mediated by Ca^2+^/Calmodulin-dependent Protein Kinase Kinase (CaMKK)-AMPK pathway^17^, so we next examined whether TMS regulated calcium signaling. To investigate whether TMS can enhance cytosolic [Ca^2+^] level, H1975 cells were stained with Fluo 3-AM to monitor and compare the cellular [Ca^2+^] level. The results of Fig. 3a demonstrated that H1975 cells displayed a remarkable increase in Fluo 3-AM fluorescence intensity upon TMS treatment right after 2 hrs.

Sarcoplasmic/endoplasmic reticulum (SERCA) is the main calcium pump that reduces the cytosol [Ca^2+^] by transferring the [Ca^2+^] to the lumen of the ER. In our study, molecular docking was carried out to explore the binding affinity of TMS with SERCA. Based on the docking score, the pose with the lowest one was selected as the most probable binding pose of TMS in SERCA. Figure 3b(A,C) illustrated that TMS could be docked into the SERCA TG binding site. The hydrophobic groups of TMS bind deeply into the hydrophobic pocket of SERCA and have favorable hydrophobic and van der Waals interactions with residues Phe256, Ile765, Ile829, Phe834 and *et al.* Compounds DMU-212 and TMS are cis-trans-isomers. However, TMS showed better and more specific inhibition effect on G-R NSCLC cells than DMU-212. To explain the reason behind this difference, we quantitatively evaluated the contribution of each residue in SERCA for TMS and DMU-212 binding and also studied the binding mode of DMU-212 to SERCA by molecular dynamics simulations. As illustrated in Fig. 3b(C,D), there were 13 and 14 residues identified as key ones (with the absolute energy contribution ≥0.5 kcal/mol) for binding TMS and DMU-212 respectively. However, binding free energy contributions of the key residues to TMS and DMU-212 are much different, which may be the origins for their different inhibition effect.

The stability of the simulation systems were assessed by the plot of structural root mean square deviation (RMSD) of protein backbone atoms versus time (supplementary Figure 3). The binding free energies of SERCA in complex with TMS and DMU-212 were calculated and the results (∆G_(MM/GBSA)) were −39.41 and −38.21 kcal/mol respectively, which is consistent well with the experimental results that TMS showed better and more specific inhibition effect than DMU-212.

Moreover, the enzyme activity of SERCA was investigated after treatment of TMS. Attractively, TMS can significantly suppress the activity of SERCA without changing SERCA expression level (supplementary Figure 4 and 5a). Therefore, from this part of result, it is clear that TMS can directly interact with SERCA and thus inhibit its activity.

To ascertain whether the increase of [Ca^2+^] levels plays an important role in TMS-induced cell death, Ca^2+^ chelator (BAPTA/AM, BM) and specific inhibitor of the CaMKK-β (STO-609) were applied to co-treat with TMS. Interestingly, Fig. 3c,d showed that both BM and STO-609 remarkably relieved the apoptosis induced by TMS. These results suggested that the increase of [Ca^2+^] level is required for TMS-induced apoptosis and suppressing the mTOR pathway.

### TMS induces endoplasmic reticulum (ER) stress in G-R NSCLC cells

**Figure 4:**
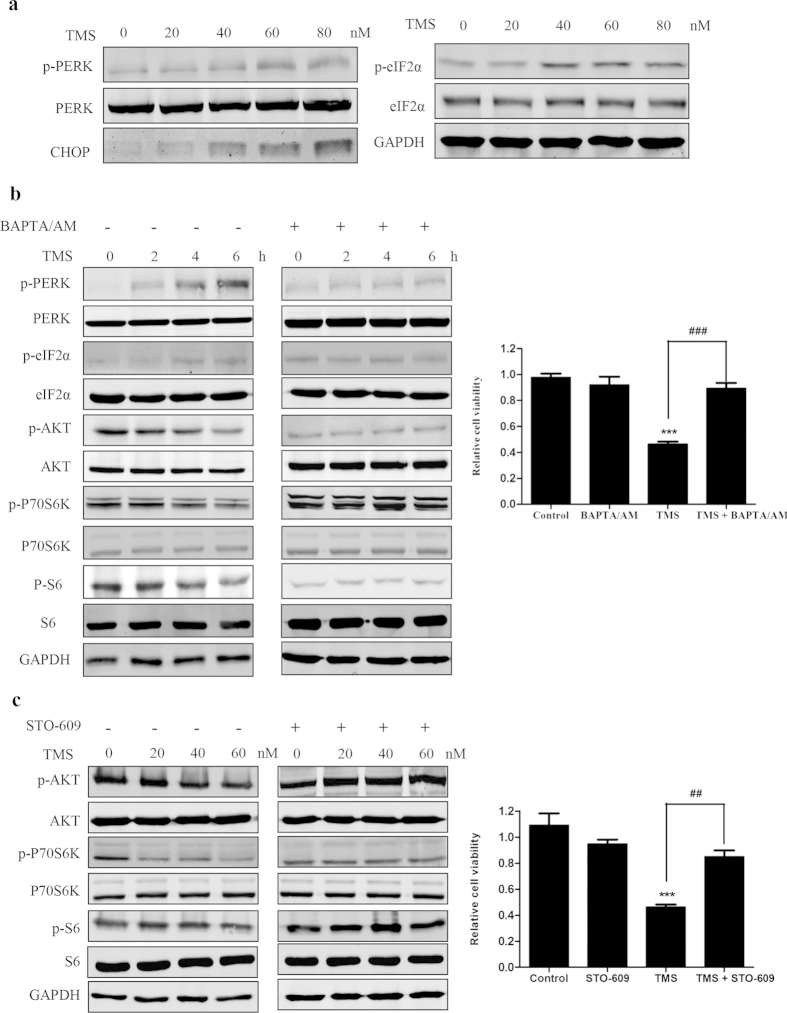
TMS induced ER stress in H1975 by mediating Ca^2+^ level. (**a**) TMS activated ER stress pathway. (**b,c**) BM and STO-609 greatly counteracted the effect of TMS on cell growth, mTOR pathway, and ER stress. (*p < 0.05, **p < 0.01, ***p < 0.001).

Calcium is essential for the activation and growth of cells, whereas overloading or prolonged cytosolic calcium elevation will lead to cells dysfunction and death, for example ER stress, autophagy and apoptosis in G-R NSCLC is yet unknown^18^. Since TMS can remarkably enhance the cytosolic [Ca^2+^] level, we further determined whether TMS can induce ER stress in H1975. As shown in Fig. 4a, the key members of ER stress pathway, PERK, eIF2α were activated and CHOP was upregulated by TMS. The result suggested that TMS significantly induced ER stress in H1975. Then, we used BM to ascertain whether the ER stress induced by TMS is due to the increase of [Ca^2+^] level. In Fig. 4b, co-treatment of BM or STO-609 with TMS almost completely blocked the effect of TMS on ER stress. Moreover, the inhibition effect of TMS on the mTOR pathway and cell growth was also counteracted by BM. Similar result was also obtained by co-treatment with STO-609 and TMS as shown in Fig. 4c.

### TMS induces AMPK activation and autophagy in G-R NSCLC cells

**Figure 5:**
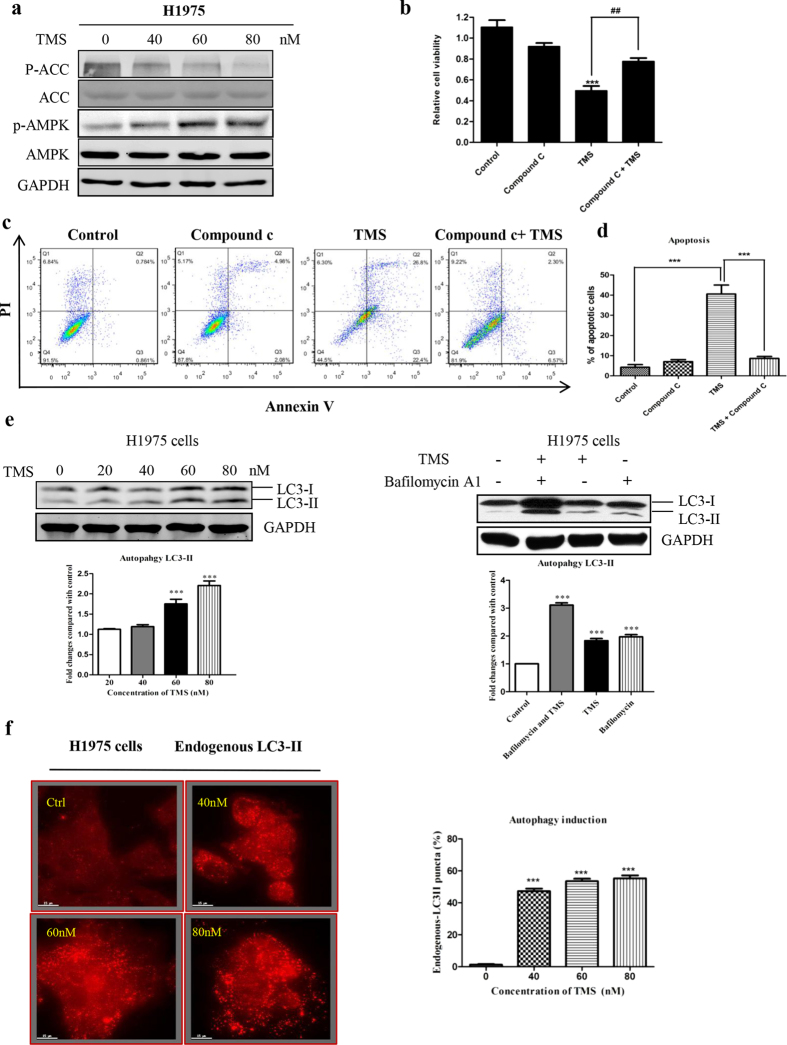
TMS activated AMPK pathway and induced autophagy in H1975. (**a**) TMS increased the phosphorylation of AMPK and its downstream target ACC. (**b,c**) Inhibition of AMPK by compound c can partially rescue the cells from apoptosis. (**d**) The statistical result of (**c**). (**e**) TMS induced autophagic LC3 puncta formation in H1975. (**f**) TMS induced autophagic flux in H1975 cells. Cells were treated with TMS (60 nM) in the presence or absence of 50 nM lysosomal protease inhibitors, bafilomycin A1 for 24 h. Cell lysates were analyzed by western blot for LC3-II conversion. (*p < 0.05, **p < 0.01, ***p < 0.001).

It is well-known that one of the important AMPK activation mechanism is by induction of calcium influx which could phosphorylate CaMKK-β and thus activate AMPK^19^. In Fig. 5a, TMS significantly induced the phosphorylation of AMPK as well as its direct downstream target acetyl-CoA carboxylase (ACC). Since AMPK is a major upstream regulator of mTOR, the activation of AMPK is able to suppress the activity of mTOR^20^. Therefore, we applied compound C which is a specific inhibitor of AMPK to block the activation of AMPK and further investigate whether it can activate mTOR and offset the effect of TMS. As shown in Fig. 5b–d, compound C partially recovered the inhibition effect of TMS on P70S6K which is the direct target of mTOR-mediated apoptosis and cell death.

In addition, activating the signaling cascade of CaMMKK-β-AMPK-mTOR can lead to autophagy^17^. In addition, autophagy is directly involved in reducing the growth of tumor cells^21^, thus, chemical autophagy inducers could have the potential of inhibiting tumor growth^22^. We used immunofluorescence to detect the LC3-II formation which is an essential protein of the autophagosome for further demonstrating if TMS induces autophagy. As shown in Fig. 5e,f, endogenous LC3-II showed a significantly increase of puncta formation in cells treated with TMS. However, this effect wasn’t observed in normal lung epithelial cells BEAS-2B (supplementary Figure 5b). On the other hand, autophagic flux can be monitored by measuring the turnover of LC3-II in the presence or absence of lysosomal inhibitors. Prevention of lysosomal degradation can be achieved through the use of protease inhibitors^23^. Thus, we determined the conversion of LC3-II by western blot in the presence of lysosomal protease inhibitors (bafilomycin A1)^23^. As expected, TMS markedly increased the rate of LC3-II formation in the presence of the inhibitors when compared with the use of either inhibitors or TMS treatment alone, suggesting that TMS induces autophagic activity due to enhanced autophagosome formation. Taken together, the above results suggested that TMS remarkably induced AMPK activation and autophagy in H1975 cells.

### JNK is involved in TMS-induced cell death

**Figure 6:**
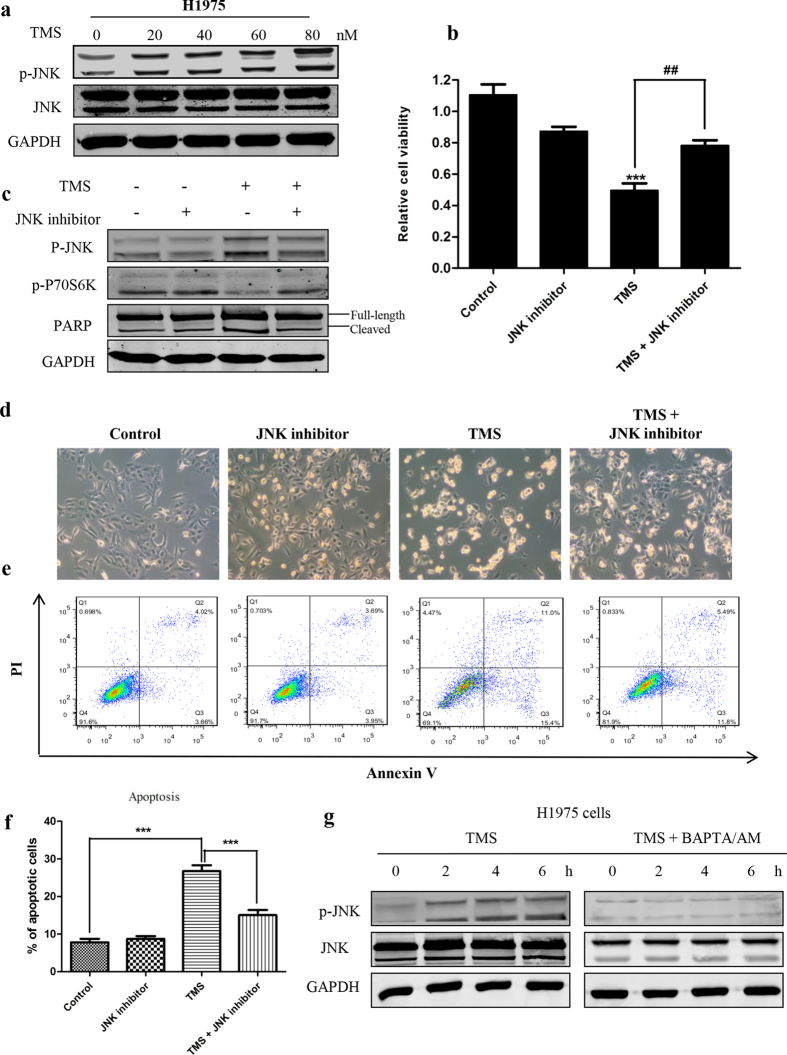
JNK activation was involved in TMS induced cell death. (**a**) TMS activated JNK. (**b**) MTT assay showed that JNK inhibitor blocked the cell death induced by TMS. (**c**) Inhibiting JNK activation can rescue the suppression of TMS on mTOR pathway. (**d–f**) Blocking the activation of JNK can greatly inhibit the apoptosis induced by TMS. (**g**) The activation of JNK was regulated by TMS through mediating [Ca^2+^] level. (*p < 0.05, **p < 0.01, ***p < 0.001).

It has been reported that c-Jun N-terminal kinases (JNK) is closely associated with ER stress and mTOR pathway^24^. To investigate whether JNK was also involved in the anti-cancer mechanism of TMS, we examined the activation of JNK with the treatment of TMS. In Fig. 6a, JNK was significantly activated by TMS. To determine whether the activation of JNK was required for TMS to induce cell death, JNK specific inhibitor SP600125 was used to inhibit the activation of JNK. Interestingly, as shown in Fig. 6b–e, the apoptosis and cell death induced by TMS were all partially rescued by JNK inhibitor. The inhibition on P70S6 kinase which is the direct downstream target of mTOR, was also largely blocked by it. These results indicated that the activation of JNK played an important role in TMS-induced cell death in gefinitib-resistant NSCLC cells.

### TMS inhibits the activation of EGFR pathway

**Figure 7:**
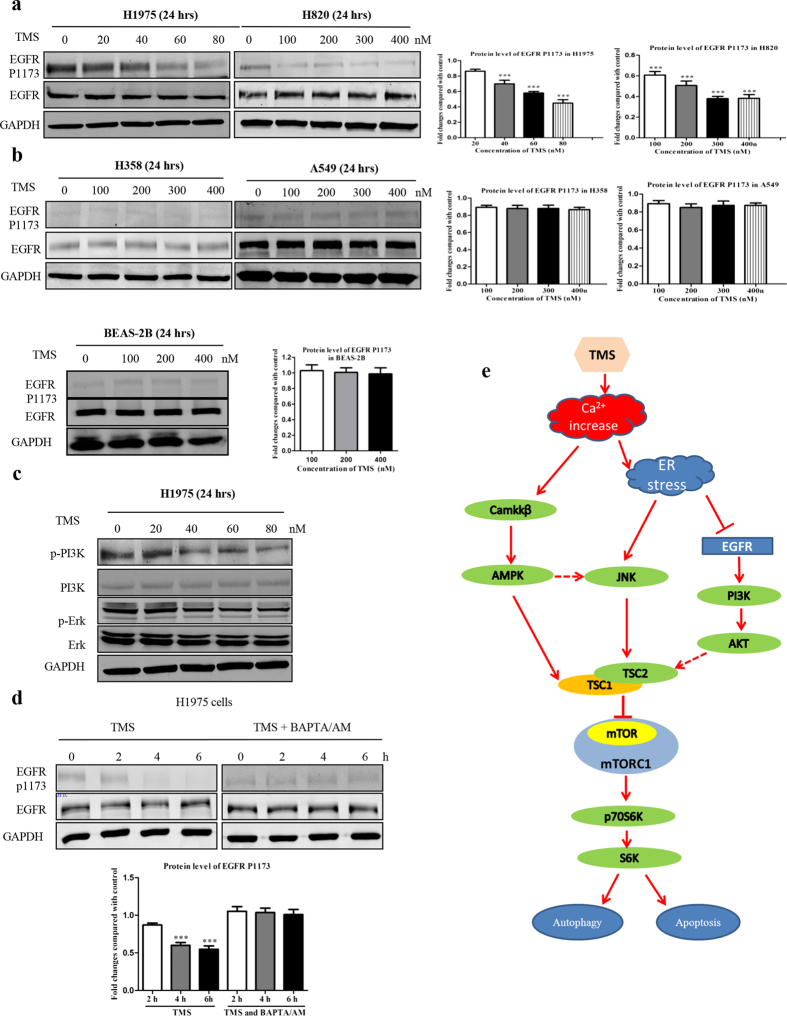
TMS inhibited EGFR activation by regulating [Ca^2+^] level. (**a**) TMS specifically inhibited the phosphorylation of tyrosine residue 1173 on EGFR in G-R NSCLC cells. (**b**) TMS has no effect on EGFR in EGFR wild-type NSCLC cells: A549 and H358 and normal lung epithelial cells BEAS-2B. (**c**) TMS also inhibited the activation of EGFR downstream pathways. (**d**) The proposed framework of TMS action on H1975 cells.

It is well-known that G-R NSCLC cells are largely dependent on constitutively active EGFR pathway. Activation of EGFR would stimulate its downstream kinases including PI3K, Akt, and mTOR^25^, and thus enhance and prolong cancer cells growth and survival. Since TMS showed discriminatory effect on G-R NSCLC cells, we investigated whether it is due to the inhibitory effect on EGFR. As shown in Fig. 7a, TMS largely inhibited the phosphorylation of EGFR on 1173 sites in two G-R NSCLC cells (H1975 and H820). Moreover, the downstream targets of EGFR, such as PI3K and ERK (as shown in Fig. 7c), were inhibited by TMS either. However, as shown in Fig. 7b, there was no significant change in both EGFR wild-type cell lines:A549 and H358; and normal lung epithelial cell line (BEAS-2B), suggesting selectively of TMS on EGFR activity across G-R NSCLC cells and EGFR wild-type cells.

To ascertain whether the inhibition of TMS on EGFR activation was mediated through the increase of intracellular calcium level, we used BM to co-treat with TMS. In Fig. 7d, BM remarkably relieved the inhibitory effect of TMS on EGFR, which indicated that the inhibition on EGFR activation by TMS is also regulated by calcium. Taken together, TMS can significantly suppress the activity of EGFR which is required for the G-R NSCLC cells for survival and growth; and contributes to the discriminatory effect of TMS.

## Discussion

Drug resistance is a Gordian knot in chemotherapy, which largely decreases the efficacy of drug treatment outcomes on patients. There are various mechanistic for inducing acquired resistance, for example, genetic mutation^26^, multi-drug resistant (MDR) genes overexpression^27^ and alternative pathway activation^28^. For G-R NSCLC patients, nearly 49% of the resistance cases are due to the presence of T790M second mutation on EGFR. Although second generation TKI has been developed, which can inhibit activation of EGFR with T790M mutation^29^. However, further acquired resistance is still unavoidably happened after prolong usage of these TKIs^30^. Therefore, developing novel targets for anti-cancer therapy, especially for G-R patients, as well as developing novel multi-targeting therapeutic strategy is urgently required.

In this study, we have firstly identified an effective agent, TMS which can effectively and specifically induce cell death in G-R NSCLC cells, whereas it is non-toxic to normal lung epithelial cells. When compared with another derivative of resveratrol, DMU-212, with IC_50_ value which falls to micro-molar level, TMS has IC_50_ value at nano-molar level and thus it is more effective than DMU-212. Also, only TMS but not DMU-212 has selectivity on G-R NSCLC cells, making it more promising with a wider therapeutic window between tumor with EGFR mutation and normal lung tissue with wild-type EGFR.

The main anti-cancer mechanism of TMS is due to regulation of calcium signaling. From our study, TMS can directly bind to SERCA and significantly increase the intracellular [Ca^2+^] level and further mediate downstream calcium signaling cascades. It has been reported that the activation of EGFR stimulated cellular calcium signaling and promoted the growth of cancer cells^31^. However, whether overloading [Ca^2+^] has negative feedback effect on EGFR is unknown. Interestingly, our study demonstrated that in G-R H1975 cells, elevation of [Ca^2+^] resulting in inhibiting of EGFR at tyrosine 1173 site, causing oncogenic shock and apoptosis in EGFR-dependent cells like H1975 and H820 but not for EGFR-independent cell line such as A549 and H358. Here, we reported at the first time, the discriminatory effect of TMS to G-R NSCLC cells could be caused by negatively feedback regulation of EGFR activation after triggering overloaded [Ca^2+^] influx.

As shown in Fig. 7e, after increasing the intracellular [Ca^2+^] level, TMS consequently caused activation of two signaling pathways, the ER stress and the CaMKK-β-AMPK- mTOR pathway. TMS significantly induced the ER stress and then apoptosis by increasing cellular [Ca^2+^] level. Moreover, ER stress triggered apoptosis through inducing JNK activation, using JNK inhibitor SP600125 largely counteracted the effect of TMS.

The result of iTRAQ proteomics also showed that TMS remarkably inhibited the CaMKKβ-AMPK- mTOR pathway. Noted that the main members of this pathway includes AKT, mTOR, P70S6K and S6^32^,^33^ which were all significantly down-regulated after TMS treatment. The mTOR signaling pathway senses and integrates a variety of environmental cues to regulate organismal growth and homeostasis^34^. It has been reported that mTOR pathway could be activated by the stimulus like growth factor, insulin and also sensing cellular nutrient, such as oxygen, and energy levels^35^,^36^,^37^. The major targets of mTOR appear to be transcriptional factors and components of the translation machinery^38^. Therefore, mTOR can directly regulate RNA transcription and translation, thus controlling cell growth and proliferation. Since mTOR is a kinase that functions as a master switch between catabolic and anabolic metabolism, it has become a hot target for the design of new anticancer agent^39^. Rapamycin is currently the most well-established mTOR inhibitors^40^. However, long-lasting objective tumor response was only shown in clinical trials, with CCI-779 being a first-in-class mTOR inhibitor that improved the survival of patients with advanced renal cell carcinoma in clinic^39^. All of these evidences further support that TMS has potential to be further explored *in vivo* and in clinics due to its strong efficacy in G-R resistant cells and converging inhibition effect on the mTOR pathway.

In sum, our study has provided new information of the potential use of a new resveratrol derivative TMS for treating G-R NSCLC patients. Since the anti-cancer mechanism of TMS is different from that of current EGFR TKIs, by not only directly targeting EGFR but exerting multi-targeting effect on EGFR as well as crosstalk pathways. Thus single use of TMS or combination use of TMS with TKIs may be beneficial to G-R NSCLC patients.

## Materials and Methods

### Materials

All chemicals and reagents were purchased from Sigma (St Louis, MO, USA) unless otherwise stated. Bafilomycin A1 was obtained from Calbiochem (San Diego, CA). TMS was purchased from ‘Great Forest Biomedical Ltd’, and the purity was confirmed with our HPLC analysis. TMS was dissolved in dimethyl sulfoxide (DMSO) to form a 100 mM stock solution, fetal bovine serum (FBS), antibiotics and RPMI medium were purchased from Gibco (Carlsbad, CA, USA). BEBM medium was purchased from Lonza (Allendale, NJ, US). RIPA lysis buffer and antibodies anti-LC3B, anti-PARP, anti-p-EGFR(Tyr845/1068/1173), anti-cleaved caspase-3 (Asp175), anti-p-extracellular signal-regulated kinase 1/2 (Erk1/2) (Thr202/Tyr204), anti-p-Akt (Ser473), anti-ribosomal protein S6 kinase (p70S6K) (Thr389), anti-PI3K, anti-JNK, anti-Erk1/2, anti-Akt, anti-p70S6K anti-S6 kinase, anti-PERK, anti-Bcl-2, and anti-EGFR were purchased from Cell Signaling Technology (Beverly, MA, USA). Antibodies anti-p-eIF2α, anti-eIF2α, anti-CHOP, anti-GAPDH and anti-SERCA were purchased from Santa Cruz (Dallas, Texas, TX, USA). Fluorescence-conjugated secondary antibodies were purchased from Odyssey (Lincoln, NE, USA). The ZyMax™ TRITC-conjugated anti-mouse secondary antibodies were purchased from Invitrogen (Scotland, UK).

### Cell culture

A549 and H1975 NSCLC cells were all purchased from ATCC. Cells were cultured in RPMI1640 medium supplemented with 10% FBS, 100 U/ml penicillin and 100 μg/ml streptomycin. The culture flasks of BEAS-2B were pre-coated with a mixture of 0.01 mg/ml fibronectin, 0.03 mg/ml bovine collagen type I and 0.01 mg/ml bovine serum albumin dissolved in BEBM medium (Lonza, Allendale, NJ, US). All the cells were cultivated at 37 °C with 5% CO_2_ incubator.

### Cell viability assay

Cells were plated in 96-well plates. After treatment of various doses of TMS for 24 h, cells viability was determined by MTT assay as described. The viability (%) was expressed as = [OD of treated group/OD of control group] × 100%. The viability of the control group was considered as 100%.

### Apoptosis analysis by flow cytometer

A hundred thousand cells were seeded in each well of 6-well plates and cultured overnight for adhesion. Next day, they were treated with a series dose of TMS for 24 h. After treatment, cells were double-stained with PI and Annexin V for 15 min. Finally, the percentage of apoptotic cells was analyzed by a flow cytometer (BD FACSAria III, BD, Franklin Lakes, NJ, USA).

### Western blot analysis

After treatment, cells were lysed in RIPA lysis buffer containing protease inhibitor cocktail for 15 min on ice and then boiled for 3 min. The protein concentration was determined with a Bio-Rad DCTM Protein Assay Kit (Bio-Rad, Hercules, CA, USA). Thirty μg protein lysate were loaded and separated by 10% SDS-polyacrylamide gel electrophoresis and transferred to a nitrocellulose (NC) membrane. The membranes were incubated with the primary antibody (1:2000) and then with a fluorescence-conjugated secondary antibody (1:10000). GAPDH was used as the loading control and for normalisation. The signal of the membranes was scanned with a LI-COR Odyssey Scanner (Belfast, ME, USA).

### iTRAQ proteomics

Cells lysate was centrifuged at 12,000 g for 5 mins at 4 °C. The supernatant was processed with ice-cold acetone and re-suspended in the dissolution buffer provided in iTRAQ Reagent 4-Plex kit (AB SCIEX, Framingham, MA, USA). The concentration of protein samples was determined by 2-D Quant Kit (GE, Fairfield, Connecticut, USA) and adjusted to 5 μg/μL.

iTRAQ labeling was carried out following the manufacturer’s protocol of iTRAQ Reagent 4-Plex kit. Briefly, the lysates were reduced with reducing reagent, alkylated with cysteine blocking reagent and digested with a trypsin to protein ratio of 1:10 (w/w) at 37 °C for 16 hr. Peptides of control group were labeled with iTRAQ reagent 116 and that of treated cells were labeled with iTRAQ reagent 117. Both samples were combined and the samples were desalted using Sep-Pak C18 cartridge (Waters, Milford, Massachusetts, USA) and then purified using a strong cation exchange (SCX)-cartridge (Phenomenex, Torrance, California, USA). The peptide sample was then applied to 24 cm pH 3.9–5.1 IPG strips for isoelectric focusing and then the IPG strips were cut into 24 equal fractions. Peptides from IPG strip fractions were extracted with a gradient solution (sequential extractions with 0.1% TFA, 50% ACN/0.1%TFA and 100% ACN/0.1% TFA). The solution of each step was vacuum dried and cleaned up by OASIS HLB cartridge (Waters, Milford, Massachusetts, USA) and eluted with 70% ACN/0.1% TFA twice. Eluates were dried under vacuum and reconstituted in 25 μL of 2% ACN/0.1% TFA before 1D-LC-MS/MS analysis.

Approximately 0.2 μg of peptides were subjected to the automated LC-MS/MS analysis, using Dionex Ultimate 3000 RSLC system coupled on-line to Bruker maXis Impact Q-TOF MS. MS/MS data was processed using Bruker Compass Data Analysis software, and the generated peak lists were submitted to MASCOT search engine against SwissProt 51.6 database.

### Computational docking

The initial structure of TMS and DMU-212 was downloaded from the PubChem (http://pubchem.ncbi.nlm.nih.gov). TMS structure was processed by the LigPrep (Version 2.3, Schrödinger, LLC, New York, NY) based on OPLS-2005 force field^41^. The ionized state was assigned by using Epik (Version 2.0, Schrödinger, LLC, New York, NY) at a pH value of 7.0 ± 2.0. The cocrystal structure of SERCA complexed with thapsigargin (TG) was retrieved from the Protein Data Bank (PDB ID code 2AGV^42^). To prepare the protein for docking, the Protein Preparation Wizard module in Schrödinger 2009 was used. TMS was docked into the TG binding site of the SERCA using the Glide (Version 5.5, Schrödinger, LLC, New York, NY) with the standard precision (SP) scoring mode. The docking grid box was defined using the Receptor Grid Generation tool in Glide by centering on TG in the SERCA. In molecular docking, 5000 poses were generated during the initial phase of the docking calculation, out of which best 1000 poses were chosen for energy minimization by 1000 steps of conjugate gradient minimizations. The best binding pose for TMS was considered for the further analysis.

### Molecular dynamic Simulations and binding free energy calculation

Molecular dynamic simulation was performed within AMBER14^42^. For each simulation, a sophisticated protocol including minimization, heating and equilibration was employed. Then, 15 ns MD simulation was conducted in NPT ensemble under a temperature of 310K and a pressure of 1 atm using a periodic boundary condition.

Binding free energy (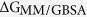) of TMS and DMU-212 to SERCA excluding entropic contribution was calculated using the single-trajectory based MM/GBSA method^43^ and given by: 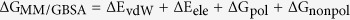


### Endogenous autophagy detection

The detection of endogenous LC3 was conducted using immunofluorescence staining method as described previously^17^. In brief, TMS-treated cancer cells on cover slips were fixed with 4% paraformaldehyde (Sigma) for 20 min at room temperature and then rinsed with PBS. Immerse coverslips in methanol at room temperature for 2 min. After washing with PBS, the cells were then incubated with anti-LC3B (1:200) in TBST (100 mM Tris HCl, pH 7.5, 150 mM NaCl, 0.05% Tween 20 and 5% BSA) overnight at 4 °C. After washing with PBS, the cells were incubated with anti-mouse secondary antibody (TRITC) 1:200 in TBST containing 5% BSA at 37 °C for 1 hr in the dark. The coverslips were then mounted with FluorSave™ mounting media (Calbiochem, San Diego, CA, USA) for fluorescence imaging and localization of LC3 autophagosomes were captured under the API Delta Vision Live-cell Imaging System (Applied Precision Inc., GE Healthcare Company, Washington, USA). To quantify autophagy, guidelines were followed to monitor autophagy^23^, the percentage of cells with punctuate LC3 immunofluorescence staining was calculated by counting the number of the cells showing the punctuate pattern of LC3 fluorescence (≥10 dots/cell) in immunofluorescence positive cells over the total number of cells in the same field. A minimum of 1000 cells from randomly selected fields were scored.

### Measurement of intracellular calcium

Changes in intracellular free calcium were measured by a fluorescent dye, Fluo-3 as previously described^44^. Briefly, H1975 cells were washed twice with culture media after TMS treatment (40–80 nM) for 4 hr. Then cell suspensions were incubated with 5 μM Fluo-3 at 37 °C for 30 min. After the cells were washed twice with HBSS, the re-suspended cell samples were then subjected to FACS analysis. At least 10,000 events were analyzed.

### The Measurement of SERCA Activity

Purified Ca^2+^ ATPase (SERCA1A) was prepared from female rabbit hind leg muscle^45^. ATPase activity was determined using the enzyme-coupled method utilizing pyruvate kinase and lactate dehydrogenase as previously described^46^. Porcine brain microsomes were prepared as described before^47^. Due to the relatively low level of Ca^2+^ATPase activity in these microsomal membranes, the rate of Ca^2+^-dependent ATP hydrolysis were measured using the more sensitive phosphate liberation assay as described before^48^. All SERCA inhibition data were fitted to the allosteric dose versus effect equation using Fig P (Biosoft, Cambridge, UK).

Activity = minimum activity + (maximum activity—minimum activity)/(1 + ([I]/IC 50) P).

### Statistical analysis

All experiments were performed in triplicate. Data were expressed as the mean ± SEM. Differences were considered statistically significant at P < 0.05.

## Additional Information

**How to cite this article**: Fan, X.-X. *et al.* (Z)3,4,5,4′-trans-tetramethoxystilbene, a new analogue of resveratrol, inhibits gefitinb-resistant non-small cell lung cancer via selectively elevating intracellular calcium level. *Sci. Rep.* **5**, 16348; doi: 10.1038/srep16348 (2015).

## Supplementary Material

Supplementary Fig 1-5

## Figures and Tables

**Table 1:** The results of proteomics analysis.

Protein accession	Description	Treat/Control
KRT84	Keratin, type II cuticular Hb4 OS	0.646
KRT85	Keratin, type II cuticular Hb5 OS	0.654
K2C1	Keratin, type II cytoskeletal 1 OS	0.54
K22E	Keratin, type II cytoskeletal 2 epidermal OS	0.629
K2C5	Keratin, type II cytoskeletal 5 OS	0.658
K2C6B	Keratin, type II cytoskeletal 6B OS	0.647
K2C75	Keratin, type II cytoskeletal 75 OS	0.656
K2C79	Keratin, type II cytoskeletal 79 OS	0.657
HGFA	Hepatocyte growth factor activator OS	0.384
CSN7B	COP9 signalosome complex subunit 7b OS	0.408
QTRD1	Queuine tRNA-ribosyltransferase subunit QTRTD1 OS	0.41
HDHD3	Haloacid dehalogenase-like hydrolase domain-containing protein 3 OS	0.412
FUND2	FUN14 domain-containing protein 2 OS	0.437
NPA1P	Nucleolar pre-ribosomal-associated protein 1 OS	0.447
VP33A	Vacuolar protein sorting-associated protein 33A OS	0.464
RM03	39S ribosomal protein L3, mitochondrial OS	0.473
GLRX2	Glutaredoxin-2, mitochondrial OS	0.475
BMS1	Ribosome biogenesis protein BMS1 homolog OS	0.489
DDI2	Protein DDI1 homolog 2 OS	0.493
BPAEA	Bullous pemphigoid antigen 1, isoforms 6/9/10 OS	0.499
IMP4	U3 small nucleolar ribonucleoprotein protein IMP4 OS	0.501
ZN556	Zinc finger protein 556 OS	0.503
SUMO1	Small ubiquitin-related modifier 1 OS	0.526
IASPP	RelA-associated inhibitor OS	0.534
APOD	Apolipoprotein D OS	0.536
PEPL1	Probable aminopeptidase NPEPL1 OS	0.54
RM16	39S ribosomal protein L16, mitochondrial OS	0.542
YTHD1	YTH domain family protein 1 OS	0.548
VIME	Vimentin OS	0.561
THIC	Acetyl-CoA acetyltransferase, cytosolic OS	0.564
EDC3	Enhancer of mRNA-decapping protein 3 OS	0.564
RL32	60S ribosomal protein L32 OS	0.57
PSMG3	Proteasome assembly chaperone 3 OS	0.574
DCAF7	DDB1- and CUL4-associated factor 7 OS	0.575
SYLM	Probable leucyl-tRNA synthetase, mitochondrial OS	0.578
PEX3	Peroxisomal biogenesis factor 3 OS	0.58
NEC1	Neuroendocrine convertase 1 OS	0.589
CHCH1	Coiled-coil-helix-coiled-coil-helix domain-containing protein 1 OS	0.591
ULA1	NEDD8-activating enzyme E1 regulatory subunit OS	0.591
SH3L1	SH3 domain-binding glutamic acid-rich-like protein OS	0.592
P5CR3	Pyrroline-5-carboxylate reductase 3 OS	0.602
NOC2L	Nucleolar complex protein 2 homolog OS	0.609
PCY2	Ethanolamine-phosphate cytidylyltransferase OS	0.61
EXOS7	Exosome complex exonuclease RRP42 OS	0.611
HEAT1	HEAT repeat-containing protein 1 OS	0.611
LCMT1	Leucine carboxyl methyltransferase 1 OS	0.611
TMSL3	Thymosin beta-4-like protein 3 OS	0.612
RCL1	RNA 3~-terminal phosphate cyclase-like protein OS	0.616
NOC4L	Nucleolar complex protein 4 homolog OS	0.617
AMPD2	AMP deaminase 2 OS	0.622
TET2	Probable methylcytosine dioxygenase TET2 OS	0.625
BRX1	Ribosome biogenesis protein BRX1 homolog OS	0.626
FRAP	Serine/threonine-protein kinase mTOR OS	0.627
ANK3	Ankyrin-3 OS	0.629
ALG13	UDP-N-acetylglucosamine transferase subunit ALG13 homolog OS	0.629
UTP15	U3 small nucleolar RNA-associated protein 15 homolog OS	0.631
CSTF1	Cleavage stimulation factor subunit 1 OS	0.633
CORO7	Coronin-7 OS	0.633
SCAM4	Secretory carrier-associated membrane protein 4 OS	0.633
KI21A	Kinesin-like protein KIF21A OS	0.634
EVPL	Envoplakin OS	0.635
RRS1	Ribosome biogenesis regulatory protein homolog OS	0.637
NHRF2	Na(+)/H(+) exchange regulatory cofactor NHE-RF2 OS	0.639
PKHD1	Fibrocystin OS	0.644
ZC3H8	Zinc finger CCCH domain-containing protein 8 OS	0.644
PTER	Phosphotriesterase-related protein OS	0.646
BL1S3	Biogenesis of lysosome-related organelles complex 1 subunit 3 OS	0.649
CHM4A	Charged multivesicular body protein 4a OS	0.65
CP088	Protein C16orf88 OS	0.65
BAX	Apoptosis regulator BAX OS	0.651
EIF1A	Probable RNA-binding protein EIF1AD OS	0.651
DLRB1	Dynein light chain roadblock-type 1 OS	0.656
NEK9	Serine/threonine-protein kinase Nek9 OS	0.657
PGTA	Geranylgeranyl transferase type-2 subunit alpha OS	0.658
TB10A	TBC1 domain family member 10A OS	0.662
TPPC5	Trafficking protein particle complex subunit 5 OS	0.663
GMDS	GDP-mannose 4,6 dehydratase OS	0.666
BIN1	Myc box-dependent-interacting protein 1 OS	0.666
CO024	UPF0480 protein C15orf24 OS	1.5
PIGS	GPI transamidase component PIG-S OS	1.504
SFRIP	SFRS2-interacting protein OS	1.507
UROK	Urokinase-type plasminogen activator OS	1.509
PRG4	Proteoglycan 4 OS	1.513
CC150	Coiled-coil domain-containing protein 150 OS	1.518
VPRBP	Protein VPRBP OS	1.522
CASP	Protein CASP OS	1.527
FNTB	Protein farnesyltransferase subunit beta OS	1.527
DCLK3	Serine/threonine-protein kinase DCLK3 OS	1.528
RNH2A	Ribonuclease H2 subunit A OS	1.531
STK25	Serine/threonine-protein kinase 25 OS	1.532
UB2R1	Ubiquitin-conjugating enzyme E2 R1 OS	1.533
PAI2	Plasminogen activator inhibitor 2 OS	1.543
CUTA	Protein CutA OS	1.546
CDK7	Cell division protein kinase 7 OS	1.547
NCOAT	Bifunctional protein NCOAT OS	1.548
RT18B	28S ribosomal protein S18b, mitochondrial OS	1.55
K0406	Uncharacterized protein KIAA0406 OS	1.55
CSN6	COP9 signalosome complex subunit 6 OS	1.551
CA050	Uncharacterized protein C1orf50 OS	1.563
TBCD	Tubulin-specific chaperone D OS	1.582
CP055	Uncharacterized protein C16orf55 OS	1.582
MAVS	Mitochondrial antiviral-signaling protein OS	1.59
JAK3	Tyrosine-protein kinase JAK3 OS	1.593
RPB1B	DNA-directed RNA polymerase II subunit RPB11-b1 OS	1.596
GNL1	Guanine nucleotide-binding protein-like 1 OS	1.6
OPRS1	Sigma 1-type opioid receptor OS	1.611
DNJC5	DnaJ homolog subfamily C member 5 OS	1.631
ECSIT	Evolutionarily conserved signaling intermediate in Toll pathway, mitochondrial OS	1.633
CE033	UPF0465 protein C5orf33 OS	1.634
ITPA	Inosine triphosphate pyrophosphatase OS	1.635
ARL1	ADP-ribosylation factor-like protein 1 OS	1.637
T2AG	Transcription initiation factor IIA subunit 2 OS	1.638
NEUL	Neurolysin, mitochondrial OS	1.65
RB3GP	Rab3 GTPase-activating protein catalytic subunit OS	1.667
VGF	Neurosecretory protein VGF OS	1.67
NUDC2	NudC domain-containing protein 2 OS	1.671
CCD12	Coiled-coil domain-containing protein 12 OS	1.683
ENL	Protein ENL OS	1.687
RASF9	Ras association domain-containing protein 9 OS	1.691
ABR	Active breakpoint cluster region-related protein OS	1.712
NDUB1	NADH dehydrogenase [ubiquinone] 1 beta subcomplex subunit 1 OS	1.718
PAG1	Phosphoprotein associated with glycosphingolipid-enriched microdomains 1 OS	1.749
PLMN	Plasminogen OS = Homo sapiens GN = PLG PE = 1 SV = 2	1.766
RAB23	Ras-related protein Rab-23 OS	1.778
OASL	59 kDa 2 ~ −5 ~ −oligoadenylate synthetase-like protein OS	1.8
GOLI4	Golgi integral membrane protein 4 OS	1.842
RM42	39S ribosomal protein L42, mitochondrial OS	1.847
SFXN4	Sideroflexin-4 OS	1.867
UAP1L	UDP-N-acetylhexosamine pyrophosphorylase-like protein 1 OS	1.872
RMD3	Regulator of microtubule dynamics protein 3 OS	1.892
FA21A	WASH complex subunit FAM21A OS	1.892
CC121	Coiled-coil domain-containing protein 121 OS	1.896
GRLF1	Glucocorticoid receptor DNA-binding factor 1 OS	1.896
MK07	Mitogen-activated protein kinase 7 OS	1.896
ZN408	Zinc finger protein 408 OS	1.896
HPS5	Hermansky-Pudlak syndrome 5 protein OS	1.918
SCOT1	Succinyl-CoA:3-ketoacid-coenzyme A transferase 1, mitochondrial OS	1.944
SNF5	SWI/SNF-related matrix-associated actin-dependent regulator of chromatin subfamily B member 1 OS	1.97
RFIP5	Rab11 family-interacting protein 5 OS	1.974
CTGF	Connective tissue growth factor OS	2.085
NACA2	Nascent polypeptide-associated complex subunit alpha-2 OS	2.101
CE022	UPF0489 protein C5orf22 OS	2.115
T2H2L	General transcription factor IIH subunit 2-like protein OS	2.149
SNX9	Sorting nexin-9 OS	2.247
CLP1L	Cleft lip and palate transmembrane protein 1-like protein OS	2.276
PPIC	Peptidyl-prolyl cis-trans isomerase C OS	2.329
PSME4	Proteasome activator complex subunit 4 OS	2.347
RRP7A	Ribosomal RNA-processing protein 7 homolog A OS	2.422
RUSD2	RNA pseudouridylate synthase domain-containing protein 2 OS	2.504
S7A6O	Protein SLC7A6OS OS	3.013
DCTN6	Dynactin subunit 6 OS	3.233
IBP5	Insulin-like growth factor-binding protein 5 OS	4.22
APOC2	Apolipoprotein C-II OS	4.812
