# Supplementary material for: (Z)3,4,5,4′-trans-tetramethoxystilbene, a new analogue of resveratrol, inhibits gefitinb-resistant non-small cell lung cancer via selectively elevating intracellular calcium level
Source: Sci Rep. 2015 Nov 6;5:16348. doi: 10.1038/srep16348 (PMC4635386; doi:10.1038/srep16348)
Supplement: Supplementary Fig 1-5 [file srep16348-s1.pdf]

Title: (Z)3,4,5,4'-trans-tetramethoxystilbene, a new analogue of resveratrol, inhibits gefitinb-resistant non-small cell lung cancer via selectively elevating intracellular calcium level

Author list: Xing-Xing Fan, Xiao-Jun Yao, Su Wei Xu, Vincent Kam-Wai Wong, Jian-Xing He, Jian Ding, Wei-Wei Xue, Tahira Mujtaba, Francesco Michelangeli, Min Huang, Jun Huang, Da-Kai Xiao, Ze-Bo Jiang, Yan-Ling Zhou, Richard Kin-Ting Kam, Liang Liu and Elaine Lai-Han Leung

Supplementary figure 1

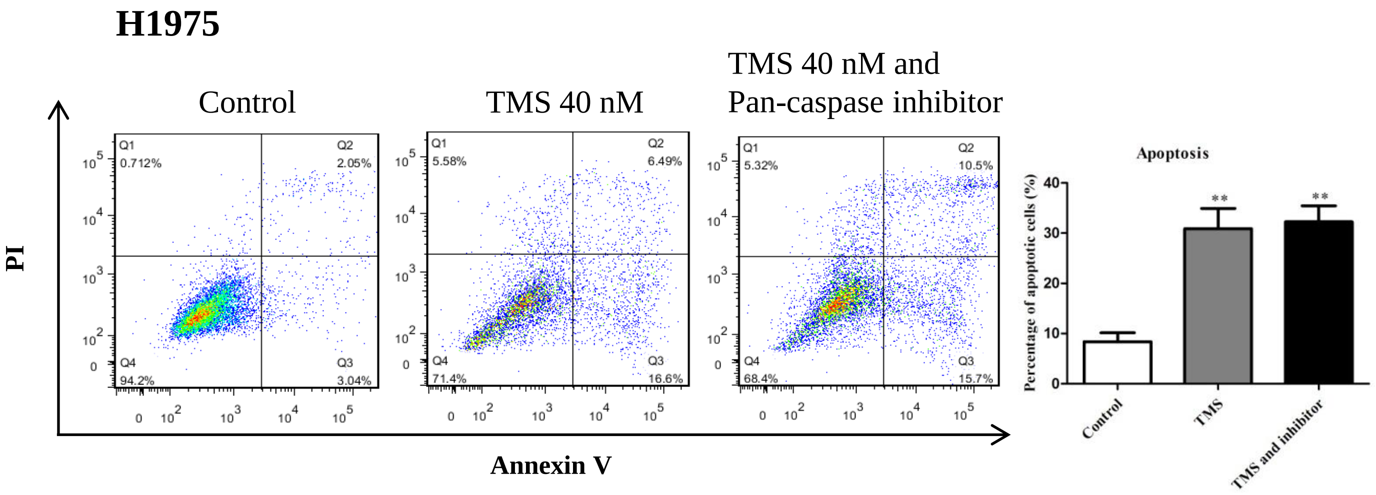

Supplementary Figure 1. Pan-caspase inhibitor failed to block the apoptotic effect on H1975 induced by TMS.

Supplementary figure 2

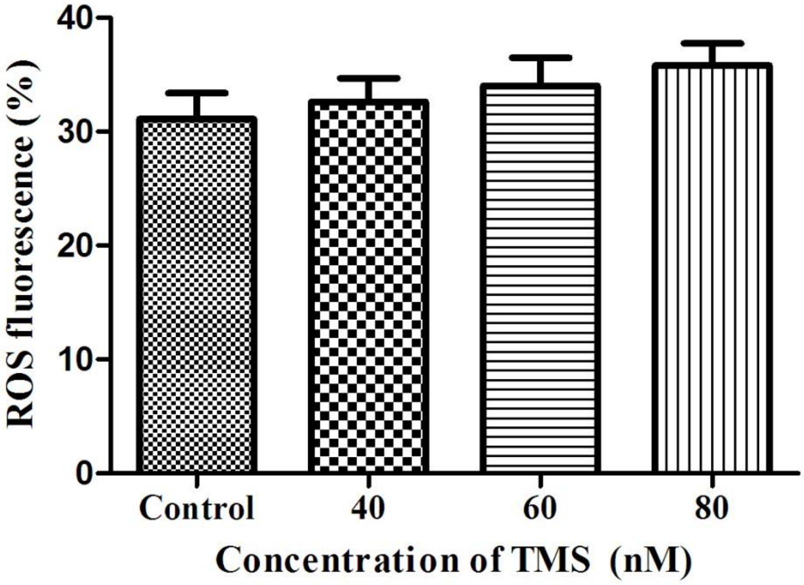

Supplementary Figure 2. TMS didn’t significantly increase the generation of ROS in H1975.

Supplementary figure 3

a

Root mean square deviations (RMSD) of heavy atoms in backbone of SERCA during Molecular Dynamic simulations.

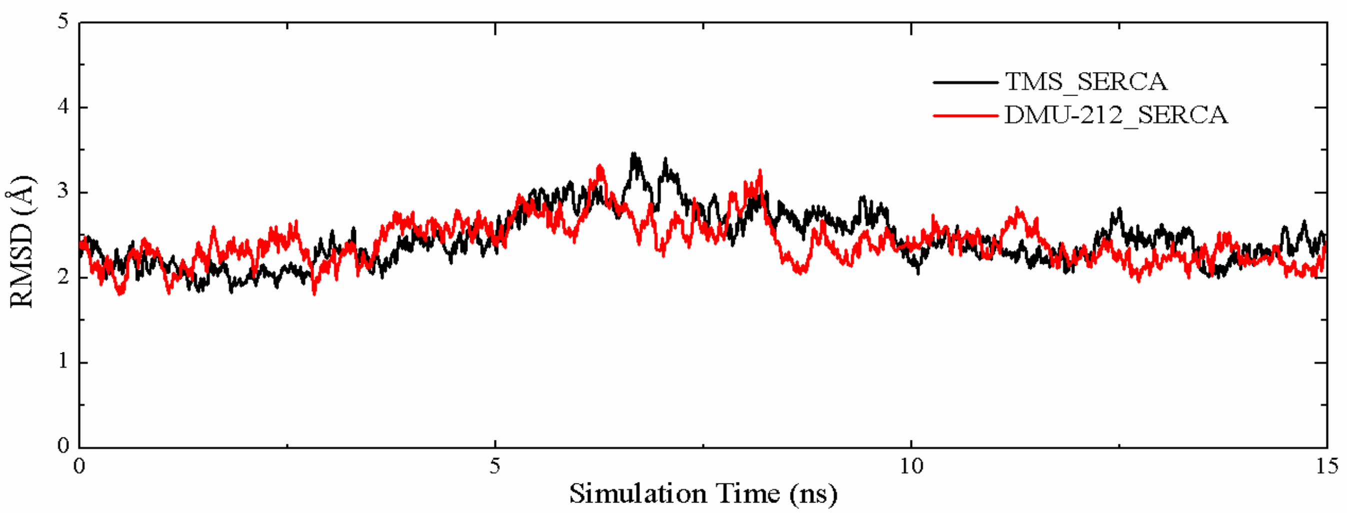

b

The calculated binding free energies of TMS and DMU-212 to SERCA (All energies are in kcal/mol)

Drugs	ΔE_{ele}	ΔE_{vdw}	ΔG_{pol}	ΔG_{nonpol}	ΔG_{MMGBSA}
**TMS**	**-4.04 ± 0.06**	**-40.27 ± 0.11**	**10.27 ± 0.06**	**-5.37 ± 0.02**	**-39.41 ± 0.12**
**DMU-212**	**-5.78 ± 0.11**	**-38.77 ± 0.10**	**11.92 ± 0.10**	**-5.57 ± 0.01**	**-38.21 ± 0.11**

Supplementary Figure 3. Molecular dynamic simulation analysis the interaction of TMS and DMU-212 with SERCA. (a) Root mean square deviations (RMSD) of heavy atoms in backbone of SERCA during molecular dynamic stimulation. (b) The calculated binding free energies of TMS and DMU-212 to SERCA (All energies are in kcal/mol).

Supplementary figure 4

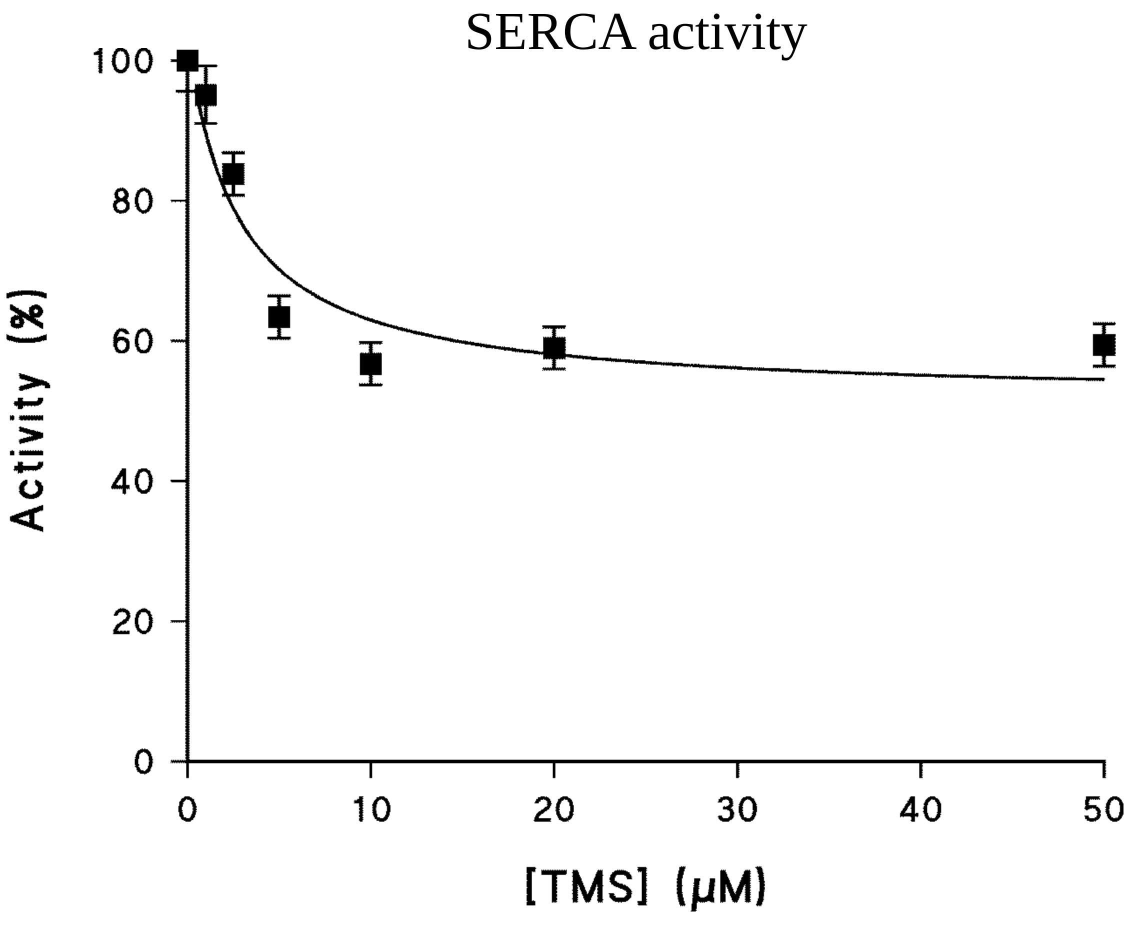

Supplementary Figure 4. TMS inhibited the enzyme activity of SERCA.

Supplementary figure 5

a

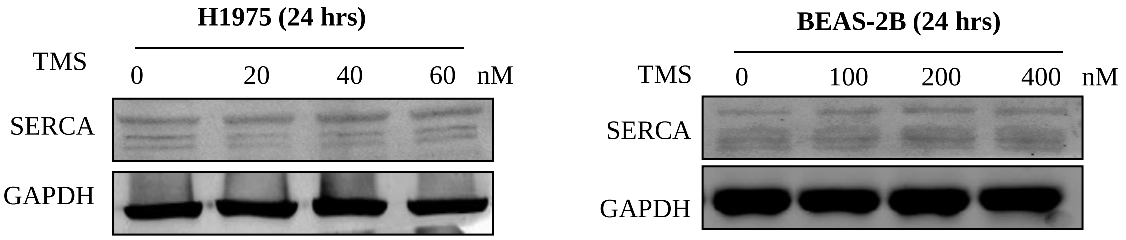

b

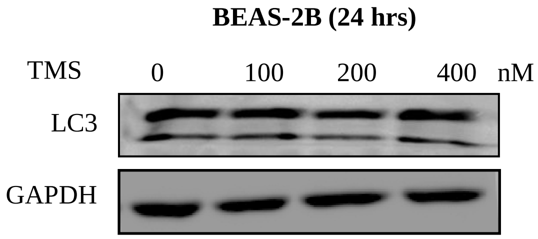

Supplementary Figure 5. TMS has no effect on SERCA expression and autophagy in normal BEAS-2B cell. (a) TMS didn't alter the expression of SERCA in both normal BEAS-2B cell and NSCLC H1975 cell. (b) TMS didn't induce autophagy in BEAS-2B. LC3-II remained the same after TMS treatment.
